# Neurosurgical enhanced recovery after surgery ERAS for geriatric patients undergoing elective craniotomy: A review

**DOI:** 10.1097/MD.0000000000030043

**Published:** 2022-08-19

**Authors:** Bolin Liu, Shujuan Liu, Tao Zheng, Dan Lu, Lei Chen, Tao Ma, Yuan Wang, Guodong Gao, Shiming He

**Affiliations:** a Department of Neurosurgery, Xi’an International Medical Center, Xi’an, China; b Department of Obstetrics and Gynecology, Xijing Hospital, Fourth Military Medical University, Xi’an, China; c Department of Neurosurgery, Tangdu Hospital, Fourth Military Medical University, Xi’an, China.

**Keywords:** cranial surgery, elderly patients, enhanced recovery after surgery, fast-track surgery, morbidity, neurosurgery

## Abstract

Population aging is an unprecedented, multifactorial, and global process that poses significant challenges to healthcare systems. Enhanced recovery after surgery (ERAS) protocols aim to optimize perioperative care. The first neurosurgical ERAS protocol for elective craniotomy has contributed to a shortened postoperative hospital stay, accelerated functional recovery, improved patient satisfaction, and reduced medical care cost in adult patients aged 18 to 65 years compared with conventional perioperative care. However, ERAS protocols for geriatric patients over 65 years of age undergoing cranial surgery are lacking. In this paper, we propose a novel ERAS protocol for such patients by reviewing and summarizing the key elements of successful ERAS protocols/guidelines and optimal perioperative care for geriatric patients described in the literature, as well as our experience in applying the first neurosurgical ERAS protocol for a quality improvement initiative. This proposal aimed to establish an applicable protocol for geriatric patients undergoing elective craniotomy, with evidence addressing its feasibility, safety, and potential efficacy. This multimodal, multidisciplinary, and evidence-based ERAS protocol includes preoperative, intraoperative, and postoperative assessment and management as well as outcome measures. The implementation of the current protocol may hold promise in reducing perioperative morbidity, enhancing functional recovery, improving postoperative outcomes in geriatric patients scheduled for elective craniotomy, and serving as a stepping stone to promote further research into the advancement of geriatric patient care.

## 1. Introduction

The world has witnessed unprecedented challenges due to an aging population and an increasing burden of healthcare. According to census data, in western countries, 1 in 5 people will be over 65 years old by 2030, and the 85-and-older will make up approximately 2% to 3% of the population. Moreover, in 2030, there will be over 200 million people over 65 years and 66 million over 80 years in China.^[1]^ Owing to the age-associated demographic trends and associated healthcare costs, there is a need to preoptimize the perioperative care of geriatric patients undergoing cranial neurosurgical procedures.

Enhanced recovery after surgery (ERAS) or fast-track surgery protocols, first conceptualized by Kehlet in 1997, have been developed rapidly and applied widely in clinical practice of multiple surgical specialties.^[2–6]^ With the optimization of perioperative care, ERAS protocols have been shown to benefit patients with shorter hospital length of stay (LOS), better postoperative functional status, lower perioperative complications, higher patient satisfaction, and cheaper healthcare costs.^[2–6]^ Not until recently, did our group propose the first neurosurgical ERAS protocol for adult patients undergoing elective craniotomy and conduct the first randomized controlled trial to validate its efficacy and safety.^[7]^ Compared with conventional perioperative care, the neurosurgical ERAS protocol is associated with shortened LOS and accelerated functional recovery.^[7]^ In the secondary analysis, decreased postoperative nausea and vomiting (PONV), absorbable skin suture, and shortened LOS are independent predictors for a higher patient satisfaction; while aspects including information transfer, professional support, shared responsibility and active participation, readiness for discharge, and follow-up are patient experience themes to be addressed to improve the quality of care.^[8]^ Furthermore, in the subgroup of glioma patients, implementation of the ERAS protocol is associated with improved health-related quality of life (i.e., higher functioning and lower symptom burden) during the follow-up of up to 6 months after surgery.^[9]^

However, these encouraging results were confined to adult craniotomy patients aged 18 to 65 years, whereas elderly patients aged >65 years were excluded. Indeed, evidence supporting the applicability and efficacy of ERAS protocols for the elderly group is mostly limited to colorectal surgery.^[10]^

Due to the lack of a neurosurgical ERAS protocol developed for geriatric patients undergoing elective craniotomy, we herein propose a novel ERAS protocol for such patients based on a review of successful ERAS protocols and optimal perioperative care of geriatric patients in the current up-to-date medical literature, in addition to our experience in implementing the ERAS protocol as a constant quality improvement program.

## 2. Methods

Similar to our previously published ERAS protocols for elective cranial and intraspinal tumor surgery,^[7,11]^ a literature review of published ERAS protocols for all surgical specialties was first performed to retrieve the key elements. Additional perioperative risk factors for complications and morbidities in geriatric patients, as well as corresponding prophylaxis and interventions, have also been identified in the literature. Panel discussions were held by the Neurosurgical ERAS Working Group, which consists of medical and ancillary staff from neurosurgery, anesthesiology, inpatient and operative nursing, and other services, including nutrition, psychiatry, physiotherapy, and rehabilitation. Key elements of the workflow were organized into 3 chronological time periods: preoperative, intraoperative, and postoperative.

## 3. ERAS protocol

### 3.1. Preoperative evaluation and management

The purpose of preoperative evaluation and management is to preoptimize geriatric patients physically, mentally, and functionally prior to the scheduled craniotomy, which includes patient and family counseling; comprehensive geriatric assessment incorporating functional, nutritional, and mental statuses; comorbidity; respiratory preparation; smoking and alcohol abstinence; antithrombotic prophylaxis; preoperative oral carbohydrate loading; antimicrobial prophylaxis; and discharge planning (Table 1). The timeline of these measures varies from weeks to 1 day prior to surgery, depending on the time required for risk assessment and intervention, as well as each individual’s medical condition. Moreover, some interventions may continue after surgery or even after discharge to maximize benefits.

**Table 1:** ERAS protocol for geriatric patients undergoing elective craniotomy.

Phase	Item	ERAS protocol
Preoperative	Patient and family counseling	Routine consultation for elective craniotomy + detailed instruction on ERAS workflow with a handbook given ≥1 wk prior to surgery
	Functional status	Assessment of functional status and physical activity + progressive exercise prehabilitation 2 to 4 wk prior to surgery
	Nutrition	Nutritional assessment and consultation for BMI <18.5 or >24, serum albumin <3.5 g/dL
	Mental status	Hospital anxiety and depression assessment + psychiatric consultation as needed
	Management of comorbidity	Appropriate preoperative assessment and specialty consultation when necessary
	Respiratory preparation	Oral and nasal cavity care: mouthwash and nasal drops
		Physical exercise: chest movement, balloon blowing, abdominal breathing exercises, cough training, respiratory muscle training
		High-risk factor (older age, past and concomitant cardiopulmonary diseases, estimated prolonged surgical time) intervention: mucolytics and expectorants
	Smoking and alcohol abstinence	Abstinence for ≥4 wk prior to surgery
	Antithrombotic prophylaxis	Active/passive limb exercise, GCS, IPC starting from admission till discharge
	Intestinal preparation	Glycerine enema for chronic constipation or no defecation ≥2 d
	Preoperative fasting and oral carbohydrate loading	Fasting period of 6 h for solids and 2 h for clear fluids
		400 mL of oral carbohydrate loading 2 h prior to surgery
	Antimicrobial skin preparation	Washing hair with chlorhexidine
		Minimal shaving a 1- to 2-cm-wide strip along the planned incision immediately before surgery
		Antibiotic prophylaxis with cefazolin within 30 min prior to skin incision
	Discharge planning	Initiate discharge planning at the time of patient education to establish expectations and ensure smooth transition home + timely and responsive follow-up strategy
Intraoperative	Microinvasive surgery	Minimally invasive approaches (e.g., keyhole surgery, endoscopic endonasal surgery), neuronavigation, electrophysiologic monitoring, hybrid operating room, awake craniotomy techniques, etc.
	Anesthetic protocol	TIVA or combined intravenous-inhalation anesthesia with short-acting agents, avoiding isoflurane
		Intraoperative use of a depth of anesthesia monitor and sedation as light as possible
	Hypothermia avoidance	Forced-air or electric heating pad + warmed fluid irrigation and infusion
	Goal-directed fluid balance	GDFR strategy + noninvasive cardiac output monitoring
	Scalp block and local incision anesthesia	Subcutaneous anesthesia with ropivacaine prior to incision and wound suturing
	Absorbable suture	Absorbable interrupted suture for dura, muscle and subcutaneous tissue, intradermal suture for skin incision, wound covered with sterile adhesive strips
	Restrictive surgical site drains	Restrict placement of surgical site drains unless deemed necessary
		If placed, remove as early as possible within 24 to 48 h
	ICU and extubation	Avoid admission to ICU whenever possible
		Extubation at the end of surgery
Postoperative	Nonopioid analgesia	Preemptive analgesia: acetaminophen the night before surgery and on the morning of surgery
		Intraoperative scalp block and local incision anesthesia + dexmedetomidine
		Postoperative repeated assessments of pain VAS scores
		Pain VAS score <4: no analgesia or oral minimal dose of nonopioid (acetaminophen, NSAIDs, and gabapentin)
		Pain VAS score 4 to 6: oral or IV nonopioid
		Pain VAS score ≥ 7: opioid ± PCA
	PONV management	PONV prophylaxis: PONV risk score ≥ 3 (assessed by PONV Simple Risk Assessment Scale), dexamethasone, 5-HT receptor antagonist (tropisetron)
		Treatment: PONV VAS ≥5, repeat 5-HT receptor antagonist (tropisetron)
		Severe or refractory cases: tropisetron + droperidol, promethazine
	Diet	Oral free fluids: 4 h after surgery
		Light diet/polymeric nutritional supplement drink: 8 h after surgery as tolerated by the patient
		Semiliquid/solid diet: 12 to 24 h after surgery
		Ordinary diet. 24 to 48 h after surgery
		Chewing gum: 3 times/d after surgery
		Immunonutrition (arginine, omega-3 fatty acids, and ribonucleotides) for malnourished patients with cancer
	Restrictive IV fluids	Rapid de-escalation of IV fluids
		Not routinely given after POD 1 unless the patient could not maintain urine output or blood pressure by oral intake
		Completely discontinued by POD 3 whenever possible
	Urinary catheter removal	Attempts to remove urinary catheter within POD 1 whenever possible
		Regular assessment of the need for maintaining the catheter
		Encouraging voiding regimens + bladder scanning + early and aggressive mobilization to prevent urinary retention
	Early mobilization and ambulation	Active management of postoperative fear of movement
		In-bed limb exercises: 6 h after surgery
		Early ambulation: POD 1
	Delirium prevention	Intraoperative use of a depth of anesthesia monitor
		Avoiding anticholinergics, antipsychotics, and benzodiazepines
		Regular assessment and nursing care
	Glycemic control	Regular monitoring with insulin treatment as needed to maintain glycemic levels 180 to 200 mg/dL
Discharge	Patient evaluation	Functional status, nutrition, mental status, anxiety and depression, pain VAS score
	Pain management	Adequate pain control with oral nonopioid
	Nutrition	Oral nutrition without IV fluids
	Vital status and healing of surgical incision	No fever or signs of infection
		Satisfactory healing of surgical incision
	Mobility	Independent mobility or mobility with minimal assistance
	Destination	Safe discharge home or to rehabilitation center
Follow-up	Home + clinic follow-up	Timely and responsive follow-up with social media cellphone/website app + outpatient clinic visit
		Functional status, pain medication use, late-onset complications, quality of life
Audit	Standardized audit and feedback	Data and safety monitoring to document process/outcome data, assess impact and encourage compliance

BMI = body mass index, GCS = graduated compression stockings, GDFR = goal-directed fluid restriction, ICU = intensive care unit, IPC = intermittent pneumatic compression, IV = intravenous, NSAIDs = nonsteroidal anti-inflammatory drugs, PCA = patient-controlled analgesia, POD = postoperative day, PONV = postoperative nausea and vomiting, TIVA = total intravenous anesthetic, VAS = visual analog scale.

#### 3.1.1. Patient and family counseling.

The importance of educating the patient and family about surgical expectations is well acknowledged, as it improves patient preparedness, satisfaction, and outcome.^[12,13]^ Detailed instruction on the ERAS protocol is necessary to offer a roadmap for the patient and family to motivate active participation. An ERAS handbook is provided at least 1 week before the scheduled surgery to allow the patient to read and ask questions.^[8]^ Additional demonstrations are provided in multiple ways (verbal, video, printed, and iPad-mediated) during hospitalization.

#### 3.1.2. Functional status.

Preoperative physical conditioning contributes to enhanced functional capacity, improved quality of life, shortened LOS, and reduced perioperative complications in patients undergoing elective orthopedic, spine, cardiac, and abdominal surgery, including the geriatric surgical population.^[14–16]^ The level of preoperative functional status could be augmented with prehabilitation, which compromises a progressive exercise program guided by physiotherapists for 2 to 4 weeks before surgery.

#### 3.1.3. Nutrition.

The patient’s preoperative nutritional status is related to perioperative morbidity and LOS, and is a modifiable risk factor to improve clinical outcomes.^[17,18]^ Although the majority of adult craniotomy patients (18–65 years) had a good nutritional status and required no nutritional intervention preoperatively,^[7]^ this may not apply to the geriatric population. Age is a well-documented risk factor for malnutrition due to physiological and anatomical changes, chronic diseases, medication use, and dietary and psychosocial habits.^[19]^ Preoperative nutritional status assessment is crucial for geriatric neurosurgical patients. Patients with a body mass index <18.5, >24, or with a low albumin level are recommended to receive nutritional consultation and intervention.^[18,19]^

#### 3.1.4. Mental status.

The patient’s mental status is also a predictor of functional outcomes after surgery.^[20]^ Therefore, preoperative evaluation of hospital anxiety and depression may help screen patients for potentially beneficial psychiatric intervention.

#### 3.1.5. Management of comorbidities.

Comorbidities such as diabetes, congestive heart failure, coronary artery disease, and chronic steroid use have been shown to increase the risk of perioperative complications in elective craniotomy patients.^[21,22]^ No single comorbidity precludes elective craniotomy, however, geriatric patients with known comorbidities should undergo appropriate preoperative assessment and specialty consultation to obtain optimal control prior to surgery.

#### 3.1.6. Respiratory preparations.

Airway risk assessment is performed with respect to age, smoking history, pulmonary function, body mass index, past and concomitant cardiopulmonary diseases, and comorbidity.^[23]^ All geriatric patients are encouraged to undergo breathing and exercise training,^[24]^ while high-risk patients should receive inhalation treatment with mucolytics and expectorants.^[23]^

#### 3.1.7. Smoking and alcohol abuse.

Both smoking and alcohol are well-recognized risk factors for postoperative complications, including difficulty in wound healing, surgical site infection, pulmonary complications, and general infections, which result in intensive care unit admission, prolonged LOS, higher reoperation rates, and greater costs.^[25,26]^ Short-term preoperative smoking cessation for 3 to 4 weeks could significantly reduce the risk of postoperative complications.^[25,27]^ Similarly, intensive preoperative alcohol abstinence for 4 to 8 weeks, which may involve pharmacological interventions for relapse prophylaxis and withdrawal symptoms, could decrease postoperative comorbidity.^[28,29]^ Therefore, all geriatric neurosurgical patients are required to abstain from smoking and alcohol for at least 4 weeks prior to surgery.

#### 3.1.8. Antithrombotic prophylaxis.

Current evidence supports the use of mechanical prophylaxis of venous thromboembolism with intermittent pneumatic compression and graduated compression stockings.^[30]^ Timing and duration of mechanical prophylaxis is critical, which should be started as early as possible preoperatively and continued until discharge to ensure maximal benefits. On the other hand, the benefits of chemoprophylaxis in reducing venous thromboembolism rates should be cautiously weighed against the increased risk of major bleeding.^[30]^ The most recent systematic review concluded that chemoprophylaxis is effective and safe in patients undergoing elective cranial or spinal surgery without increasing major or minor bleeding events.^[31]^ Yet, the robustness of the conclusion for the elderly subpopulation and the optimal time of chemoprophylaxis remain unvalidated and warrant further investigation. Owing to safety concerns, patients in China are instructed and monitored to receive mechanical prophylaxis starting from admission until discharge.

#### 3.1.9. Preoperative oral carbohydrate loading.

Instead of traditional prolonged fasting, modern guidelines advocate a shortened period of preoperative fasting, with the allowance of clear liquids up to 2 hours and solids up to 6 hours prior to surgery.^[32]^ Ample evidence has proved the role of preoperative carbohydrate loading in attenuating metabolic stress response (e.g., insulin resistance and protein breakdown) and improving clinical outcomes (e.g., pulmonary function, handgrip strength, and LOS) without increasing complication rates.^[33–35]^ Additionally, no difference in complication rates, peak glycemic levels, or insulin requirements is found between diabetic patients who receive and those who do not receive preoperative carbohydrates.^[36]^ For geriatric patients without gastrointestinal mobility disorder, preoperative oral carbohydrate is integrated into the routine procedure.

#### 3.1.10. Discharge planning.

A predefined discharge criterion is used for patients undergoing elective neurosurgery, which includes adequate pain control, adequate oral nutrition, no fever, independent mobility, and a safe discharge destination.^[7,11]^ Besides following the robust criteria, an appropriate timing for discharge planning is of vital importance to secure early discharge in the ERAS protocol and improve patient satisfaction.^[8,37]^ Therefore, discharge planning is initiated at the time of patient education to establish patient and family expectations and to increase their sense of security on early discharge, which also includes the availability of a variety of services and benefits upon discharge from the inpatient stay. A timely and responsive follow-up strategy could enhance patient experience through improved communication and access.^[8]^ Furthermore, discharge planning and home follow-up in hospitalized elders could contribute to fewer readmissions and lower costs.^[38]^

### 3.2. Intraoperative management

Intraoperative management primarily focuses on the day of surgery, the goal of which is to optimize surgical and anesthetic procedures and minimize stress response. The associated measures included microinvasive surgery, anesthetic protocol, hypothermia avoidance, goal-directed fluid balance, scalp incision anesthesia, absorbable sutures, and restrictive drains (Table 1).

#### 3.2.1. Microinvasive surgery.

The concept of microinvasive surgery involves not only less invasive approaches (e.g., keyhole surgery and endoscopic endonasal surgery),^[39]^ but also maximal neuroprotection and functional preservation with the aid of multimodal neuronavigation, electrophysiologic monitoring, awake craniotomy, and intraoperative MRI. Surgery duration and estimated blood loss are established risk factors for postcraniotomy complications in the elderly.^[21,40]^ Neurosurgeons and anesthesiologists should make efforts to minimize the length of the procedure and blood loss.

#### 3.2.2. Anesthetic protocol.

For neurosurgical patients, though no significant difference is found between total intravenous anesthetic (TIVA) and inhalational anesthetic of sevoflurane regarding time to emergence from anesthesia and brain relaxation, TIVA seems to be moderately preferable in terms of a lower risk of PONV.^[41–43]^ Use of isoflurane in craniotomy patients is not recommended due to delayed emergence from anesthesia and decreased brain relaxation.^[41]^ TIVA or combined intravenous (IV)-inhalation anesthesia with short-acting agents could be applied according to institutional practice patterns and anesthetist preference.

#### 3.2.3. Hypothermia avoidance.

Inadvertent perioperative hypothermia is a common and detrimental phenomenon that is associated with prolonged postanesthetic recovery and postoperative complications, including surgical site infection, delayed wound healing, pressure ulcer, bleeding, and cardiovascular events.^[44,45]^ Active warming has a beneficial effect in reducing complication rates, blood transfusion need, and medical costs.^[44,45]^ Awareness of the consequences of hypothermia is indispensable, and avoiding hypothermia is crucial for all surgical patients. According to ERAS protocols, forced-air or electric heating pads, together with warmed irrigation and IV fluids, are applied to all neurosurgical patients.^[7,11]^

#### 3.2.4. Goal-directed fluid balance.

During cranial surgery, anesthesiologists face the dilemma of restricting IV fluids to prevent brain swelling and maintain hemodynamic stability. To meet the dual goals of satisfactory exposure and hemodynamic stability, strategies for cardiac output-guided hemodynamic management and fluid balance with intraoperative goal-directed fluid restriction have been explored, which are associated with decreased intensive care unit stay, costs, and postoperative complications.^[46,47]^ This strategy has become standard practice with consistent implementation of neurosurgical ERAS protocols,^[7,11]^ and is applicable to geriatric patients.

#### 3.2.5. Local incision anesthesia.

Placement of the pin head holder and scalp incision are the most painful stages of craniotomy. Scalp block or local incision anesthesia can reduce the hemodynamic response to painful stimuli, as well as postoperative pain, analgesic consumption, and PONV.^[7,11,48,49]^ These are already routine procedures in several centers and are mandatory for all geriatric neurosurgical patients.

#### 3.2.6. Absorbable suture.

In addition to achieving satisfactory wound healing, absorbable sutures offer cosmetic advantages and reduce discomfort of suture removal.^[50,51]^ Absorbable sutures are a key component of our ERAS protocols and are associated with a decreased postoperative LOS.^[7,11]^ Dura, muscle, and subcutaneous tissue are closed with interrupted absorbable sutures, and the skin incision is closed with an intradermal running suture.

#### 3.2.7. Restrictive surgical site drainage.

Avoiding surgical site drainage is another distinguishing component of the ERAS protocol. If drain placement is deemed necessary by the chief surgeon, it is removed as early as possible after surgery, mostly within 24 to 48 hours. This measure is safe and effective in reducing postoperative discomfort associated with drains, promoting early ambulation, and shortening the LOS without increasing the rate of surgical complications.^[7,11]^

### 3.3. Postoperative management

Postoperative management aims to reduce postoperative morbidity and maximize patients’ physical conditioning to promote functional recovery after surgery. This consists of nonopioid analgesia, PONV management, postoperative diet, restrictive IV fluids, urinary catheter removal, early ambulation, delirium prevention, and glycemic control (Table 1).

#### 3.3.1. Nonopioid analgesia.

The incidence, magnitude, and duration of postoperative pain experienced by craniotomy patients have been underestimated for decades, and undertreated pain remains a problem.^[52]^ What is worse, acute postoperative pain becomes chronic pain in approximately 20% to 60% of patients after craniotomy.^[52]^ Patient-controlled analgesia (PCA) with morphine is an effective method in craniotomy patients, however, there are risks of PONV, sedation, respiratory depression, ileus, urinary retention, and cardiovascular events, especially in geriatric patients.^[52–54]^ In our previous neurosurgical ERAS protocols, the traditional regimen of postoperative morphine PCA is abandoned, instead, we have applied a standardized and nonopioid analgesia strategy based on repeated assessments of pain visual analog scale (VAS) scores.^[7,11]^ No analgesia or minimal oral nonopioid is prescribed for patients reporting mild pain (VAS score 1–3); regular oral or IV nonopioid is given in cases of moderate pain (VAS score 4–6); opioid is reserved for patients with severe pain (VAS score 7–10) while morphine PCA is only used in refractory cases.^[7,11]^ This strategy has achieved satisfactory pain control with significantly decreased opioid consumption and PCA use. The shift from morphine PCA to nonopioid analgesia can not only mitigate the potential side effects of opioids, such as nausea, sedation, and dizziness, but also enhance postoperative mobility.^[55]^ Additionally, local incision anesthesia and active management of PONV also contribute to improved pain control.^[7,55]^ As an adjunct to general anesthesia, intraoperative dexmedetomidine, ketamine, and lidocaine have been used for acute postcraniotomy pain control.^[12]^ Of note, Dexmedetomidine is associated with reduced PONV in addition to reduced postoperative pain and analgesic consumption.^[56]^ Lastly, preemptive analgesia with medications such as nonsteroidal anti-inflammatory drugs, acetaminophen, and gabapentinoids is another strategy to prevent postoperative pain by inhibiting autonomic hyperactivity, which also reduces PONV.^[13,57,58]^ Taken together, a multimodal nonopioid analgesia strategy as a continuum pre-,intra-,and postoperatively is proposed for geriatric neurosurgical patients.

#### 3.3.2. PONV management.

The high prevalence of PONV in craniotomy patients (ranged 20%–70%) and the increased risks of intracranial bleeding, brain edema, and aspiration associated with PONV signify the importance of appropriate PONV management, which consists of perioperative risk assessment, management of surgical and anesthesia-related risk factors, and multimodal pharmacological interventions targeting different chemoreceptors in the vomiting center.^[42,59]^ The most effective and commonly used medications are 5-HT3 antagonists, glucocorticoids, NK1 receptor antagonists, or their combination.^[42]^ According to our previous successful ERAS protocols,^[7,11]^ active PONV prophylaxis with dexamethasone and tropisetron is given to patients with a PONV risk score≥3 according to the PONV Simplified Risk Assessment Scale.^[60]^ Postoperative treatment with repeated tropisetron is given to patients reporting a PONV VAS score ≥5, and a combination of tropisetron and droperidol, or promethazine is given to refractory cases.^[7,11]^

#### 3.3.3. Postoperative diet.

Early restoration of oral nutrition is a foundation of all ERAS protocols.^[61]^ Given the nature of cranial surgery where the operative site is independent of the gastrointestinal tract, as well as the use of nonopioid analgesia and active PONV management, early diet advancement is encouraged for all craniotomy patients except for those remaining comatose for a prolonged period.^[7,12]^ Oral free fluids are permitted as early as 4 hours after surgery; light diet or a polymeric nutritional supplement drink is given 8 hours after surgery, semiliquid/solid diet is allowed 12 to 24 hours after surgery, and restoration of ordinary solid diet is achieved within 24 to 48 hours after surgery.^[7,11]^ This strategy is well tolerated by the neurosurgical patients and is associated with improved functional status compared with the conventional perioperative care which roughly doubles time to diet advancement.^[7,11]^ In addition, chewing gum is an convenient, economical and harmless way for amelioration of postoperative ileus and recovery of gastrointestinal function.^[62]^ In keeping with the evidence, an early diet advancement regimen combined with chewing gum is provided for geriatric neurosurgical patients. Supplementary immunonutrition should be considered for malnourished patients with cancer.^[12,13]^

#### 3.3.4. Urinary catheter removal.

Prolonged use of urinary catheters is associated with an increased risk of urinary tract infection in surgical patients.^[63]^ Considering the preventable nature of hospital-acquired urinary tract infection, evidence-based preventive intervention is to remove the urinary catheter on postoperative day (POD1) or as early as possible according to daily assessment of the need for maintaining the catheter.^[12,63]^ Early removal of the urinary catheter within 6 hours is feasible in neurosurgical patients, which promotes mobility and shortens postoperative LOS without increasing the rates of urinary retention.^[7,11]^ Moreover, implementing a 2- to 3-hour voiding schedule immediately after catheter removal, using bladder scanning, and encouraging early and aggressive mobilization may help to decrease the risk of urinary retention. Attempts to remove the urinary catheter within POD1 are recommended for geriatric neurosurgical patients, and regular assessment is required to maintain the catheter to ensure removal as early as possible.

#### 3.3.5. Early mobilization and ambulation.

Early mobilization and ambulation is an integral and proven strategy to prevent muscle loss, improve cardiopulmonary function, and reduce deep vein thrombosis risk and insulin resistance.^[5,64,65]^ Adherence to early mobilization and reduction in associated complications are keys for successful ERAS protocols.^[65]^ With adequate pain control and PONV management, off-bed ambulation is achievable for neurosurgical patients on POD1.^[7,11]^ Postoperative fear of movement is addressed and actively management by the doctors, nurses, physiotherapists and psychiatrists. Patients were instructed and monitored by physiotherapists to start in-bed limb exercises 6 hours after surgery and ambulation within 24 hours after surgery.

#### 3.3.6. Delirium prevention.

Given the high prevalence of postoperative delirium in the elderly and the lack of first-choice treatment, prevention is the best strategy. Intraoperative use of a depth of anesthesia monitor and sedation as light as possible are recommended.^[66]^ Additionally, avoiding medications such as anticholinergics, antipsychotics, and benzodiazepines whenever possible is vital in elderly patients, considering the risk of causing/worsening dementia, delirium, and falls.^[54]^ Furthermore, ERAS components of prehabilitation, early oral feeding, early mobilization, restrictive use of drains and invasive tubes, as well as regular assessment and nursing care all contribute to a lower risk of delirium.^[67,68]^

### 3.4. Outcome measures

Outcome measures included objective and subjective patient-reported outcomes (Table 2). Hospital LOS is the most commonly used outcome measure in ERAS protocols as a proxy for functional status and a key criterion for evaluating a successful ERAS protocol.^[65,69]^ Other objective outcomes include postoperative morbidity and mortality, surgical and nonsurgical complications, reoperation and readmission rates, functional recovery status (such as pulmonary function, muscle strength, nutritional parameters, diet advancement, and mobility), analgesic consumption, and healthcare costs.

**Table 2:** Outcome measures.

Outcomes	Parameters, assessment technique/instrument
Objective outcomes	
Hospital LOS	
Postoperative complications	Surgical complications (e.g., SSI, intracranial infection, seizure, and hemorrhage) and nonsurgical complications (e.g., cardiovascular, respiratory, gastrointestinal complications, UTI, DVT, and VTE)
Mortality	
30-d reoperation	
30-d readmission	
Functional recovery status	Pulmonary function (vital capacity and peak expiratory flow rate)
	Cardiovascular function (treadmill performance)
	Muscle strength (handgrip strength)
	Nutrition (BMI, serum albumin, and body composition)
	Time to tolerate diet (first water intake and oral liquid/solid food)
	Time to remove urinary catheter
	Mobility (time to ambulation, pedometer, and independent mobility)
	Overall functional status (KPS score)
Analgesia consumption	Opioid versus nonopioid
Healthcare cost	
Patient-reported outcomes	
Pain	VAS score
PONV	VAS score
Fatigue/frailty	VAS score, FRAIL Scale
Anxiety and depression	Hospital Anxiety and Depression Scale
General health perceptions and quality of life	SF-36, EORTC QLQ-C30/BN20
Patient satisfaction and comfort	Appropriate and validated questionnaire

DVT = deep vein thrombosis, EORTC QLQ-C30/BN20 = European Organisation for Research and Treatment of Cancer Quality of Life Core Questionnaire 30/Brain Cancer Module, KPS = Karnofsky performance status, LOS = length of stay, PONV = postoperative nausea and vomiting, SSI = surgical site infection, UTI = urinary tract infection, VAS = visual analog scale, VTE = venous thromboembolism.

Patient-reported outcomes have been suggested as the gold standard for assessing the clinical efficacy of surgical interventions and patient-perceived quality of care, and thus are preferable outcome measures for ERAS protocols.^[8,18,65,69]^ The most frequently used and important outcomes are patient-reported symptom status outcomes, such as pain, PONV, fatigue, anxiety and depression, general health perceptions and quality of life, and patient satisfaction and comfort.

## 4. Discussion

With the successful development and implementation of the neurosurgical ERAS protocol for elective neurosurgical patients in clinical trials,^[7,8,11]^ the proven benefits of reducing LOS, accelerating postoperative functional recovery, and improving patient satisfaction have called into attention the constant and universal application of the ERAS protocol in everyday practice. Compared with other surgical specialties, neurosurgery, especially cranial surgery, lags behind in the era of ERAS, and perioperative care continues to resemble conventional methods in many centers.^[5,6,18]^ As the most populous country in the world, China has the largest aging population, which is confronting healthcare providers with greater challenges in managing multiple comorbidities associated with advanced age and improving the quality of perioperative care. This multimodal, multidisciplinary, and evidence-based ERAS protocol for geriatric patients undergoing elective craniotomy aimed to establish a feasible, applicable, reasonable, and standard pattern of practice that involves and engages healthcare providers from multiple services. Under the coordination and cooperation of the Neurosurgical ERAS Working Group, cumulative benefits contributed by individual components of the ERAS protocol may translate into significant improvements in patient recovery after cranial surgery. Moreover, given the multistep nature of an ERAS protocol, both patient and provider compliance are decisive factors for success or failure. A systemic audit of process adherence and outcomes is a useful tool for assessing impact and enhancing compliance.^[7,11,12]^

## 5. Conclusion

The current proposal for a multimodal, multidisciplinary, and evidence-based ERAS protocol for geriatric patients undergoing elective craniotomy is feasible and crucial for optimizing perioperative care in this patient population. This approach is generalizable from the previous accomplishment of a neurosurgical ERAS protocol for adult patients ages 18 to 65 years and tailored to the geriatric population. Implementation of the current protocol may hold promise in reducing perioperative morbidity, enhancing functional recovery, shortening LOS, and serving as a stepping stone to guide future efforts to improve geriatric patient care.

## Author contributions

**Conceptualization:** Bolin Liu, Shiming He, Shujuan Liu.

**Data curation:** Bolin Liu, Dan Lu, Lei Chen, Shiming He, Shujuan Liu, Tao Ma, Tao Zheng, Yuan Wang.

**Formal analysis:** Bolin Liu, Shiming He, Shujuan Liu, Tao Zheng.

**Funding acquisition:** Bolin Liu.

**Supervision:** Guodong Gao, Shiming He.

**Writing – original draft:** Bolin Liu, Shujuan Liu.

**Writing – review & editing:** Bolin Liu, Dan Lu, Guodong Gao, Lei Chen, Shiming He, Shujuan Liu, Tao Ma, Tao Zheng, Yuan Wang.
